# Immunology of Bats and Their Viruses: Challenges and Opportunities

**DOI:** 10.3390/v6124880

**Published:** 2014-12-08

**Authors:** Tony Schountz

**Affiliations:** Arthropod-borne and Infectious Diseases Laboratory, Department of Microbiology, Immunology and Pathology, College of Veterinary Medicine, Colorado State University, Fort Collins, CO 80524, USA; E-Mail: tony.schountz@colostate.edu; Tel.: +1-970-491-7350; Fax: +1-970-491-8326

**Keywords:** chiroptera, reservoir host, virus ecology, zoonosis, emerging infectious disease, RNA-seq, metabolomics

## Abstract

Bats are reservoir hosts of several high-impact viruses that cause significant human diseases, including Nipah virus, Marburg virus and rabies virus. They also harbor many other viruses that are thought to have caused disease in humans after spillover into intermediate hosts, including SARS and MERS coronaviruses. As is usual with reservoir hosts, these viruses apparently cause little or no pathology in bats. Despite the importance of bats as reservoir hosts of zoonotic and potentially zoonotic agents, virtually nothing is known about the host/virus relationships; principally because few colonies of bats are available for experimental infections, a lack of reagents, methods and expertise for studying bat antiviral responses and immunology, and the difficulty of conducting meaningful field work. These challenges can be addressed, in part, with new technologies that are species-independent that can provide insight into the interactions of bats and viruses, which should clarify how the viruses persist in nature, and what risk factors might facilitate transmission to humans and livestock.

## 1. Introduction

Bats belong to Chiroptera, the second-most species-rich order of mammals with more than 1100 species [1]. Genomic evidence suggests that bats share most features of other mammals [2,3,4]; however, they have other anatomic and behavioral features that appear to be unique and some of those may be essential for hosting infectious agents. In particular, the sizes of colonies (sometimes in the millions), torpor of some species, their mutual grooming habits, gregarious social behavior, and population densities likely facilitate the rapid spread of infectious agents among bats [1,5,6,7,8]. Infection of any organism has an inherent cost to the host and infectious agent, and a balance of host response and virus replication is essential for establishment of a reservoir host/virus relationship. Thus, it is likely that bats and their viruses have co-adapted in a relationship that limits disease, but also impairs antiviral responses.

Bats also are susceptible to infectious diseases that can have dramatic impacts on their health and ecology. White nose syndrome, caused by the fungus *Pseudogymnoascus destructans*, has killed millions of bats in eastern North America, threatening some species with extinction [9,10,11]. *Bartonella* species have been isolated from many bat species [12,13,14,15,16], although the impact of these bacteria on human or veterinary health is unclear. Several other viral, bacterial, protozoal, nematode and trematode species have been associated with disease and deaths of bats. Herpesviruses, *Pasteurella* spp., *Salmonella* spp., *Yersinia pseudotuberculosis*, *Burkholderia* spp., *Cedecea davisae* and *Clostridium sordellii* have been isolated from or detected in dead bats [17,18,19]. Artibeus bats are naturally susceptible to *Histoplasma capsulatum*. Experimental infection of *A. literatus* with *H. capsulatum* resulted in fatal histoplasmosis in some bats but chronic systemic infection in others, suggesting this species can act as a reservoir but also develop disease [20], similar to what is observed in rabies virus infections of bats.

Two important features of bats that are unique among mammals are powered flight, which permits large home ranges, including over large bodies of water that can be transited in relatively short amounts of time, and nocturnal activity [21]. These features present difficulties for studying bats and their infectious agents under field conditions. Ideally, closed laboratory colonies of bats should be developed to facilitate model development, but such colonies are difficult to establish because of the logistics of capturing, quarantining and transporting bats (often to other hemispheres), the expense of adequate facilities for breeding, the knowledge and expertise of veterinary care staff, and the low fecundity of bats. Together, these features make quality bat infectious disease research difficult and expensive.

Because of these limitations, most studies on the relationships of bats and their viruses rely upon virological and serological testing of natural populations of bats. This presents several difficulties in interpreting data because it is frequently impossible to know when a given bat was infected, if it was infected with other agents that might confound interpretation of data, or if hormones or environmental stressors were influencing immune responses.

Although more than a hundred viruses have been detected in, or isolated from, bats [1,22,23,24,25,26,27,28,29], only a few bat species have been identified that are reservoirs or suspected reservoirs of infectious agents that have high impact on human health (Table 1). Nonetheless, the large population sizes of bats, hunting of bats as food sources, agricultural and livestock practices, and encroachment of humans upon bat habitat will likely lead to continued spillover events and outbreaks of disease [30,31].

**Table 1:** High impact viruses and their (suspected) bat reservoir hosts.

Virus	Disease	Reservoir Host
Rabies virus and other lyssaviruses [1,32]	Rabies	Many bat species, world-wide distribution
Marburg virus [33]	Marburg virus disease	Egyptian fruit bat (*Rousettus aegyptiacus*)
Ebolaviruses [34]	Ebola virus disease	Hammer-headed bat (*Hypsignathus monstrosus*), Franquet’s epauletted fruit bat (*Epomops franqueti*), little collared fruit bat (*Myonycteris torquata*) ^1^
SARS-CoV [26]	Severe acute respiratory syndrome	Chinese horseshoe bat (*Rhinolophus* spp.) ^2^
MERS-CoV [35]	Middle East respiratory syndrome	Egyptian tomb bat (*Taphozous perforatus*) ^2^
Nipah and Hendra viruses [36,37]	Encephalitis	Certain flying foxes (*Pteropus* spp.)
Sosuga virus [38]	Severe Acute Febrile Disease	*Rousettus* spp. ^1^

^1^ Suspected reservoirs; ^2^ Nucleotide sequences found with high similarity to SARS or MERS coronaviruses.

As is usual with reservoir/virus relationships, viruses must: (a) infect and persist in their bat hosts without causing substantial disease; (b) be transmitted to other susceptible hosts before the immune response eliminates the infection; or (c) the host dies from the infection [1]. For these reasons, many microbial agents, particularly viruses, have evolved immune-evasion strategies that manipulate the host response in a manner favorable to the virus [39,40,41,42]. These immune evasion molecules, most of which are virally-encoded accessory proteins, also affect other species, such as humans, after spillover events. However, because the molecules are evolutionarily-optimized for the reservoir hosts, they likely behave differently in the nonreservoir hosts and may contribute to pathogenesis. Nearly all laboratory work examining immune-modulating proteins from bat viruses is performed with immortalized rodent, human or non-human primate cell lines. The difficulty in interpreting these data is that no comparisons have been made to the reservoir host cells. Nipah virus P, V and W proteins target the STAT1 pathways in human cells [43], but these viral proteins have not been examined in cells from *Pteropus giganteus*, a natural reservoir of Nipah virus. If they behave identically in humans and bats, their roles in pathogenesis of human disease is suspect.

Experimental expression of viral proteins in mammalian cells often uses viral genes cloned into mammalian expression vectors that produce abundant viral protein that is probably not reflective of the levels found in cells infected with virus [42,43,44,45]. This presents another interpretation difficulty because host responses of reservoirs can be fine-tuned by evolution and subtle quantitative changes in gene expression can dramatically impact infectious outcomes [46]. It may be that activities of these viral proteins are quantitatively different in cells from reservoir hosts than what occurs in human cells, and these differences may explain why humans develop disease whereas the reservoir does not. Experiments examining the functions of these proteins should use vectors that have controllable promotors, such as those mediated by tetracycline, so that quantitative effects can be measured and compared between primary bat cells and cells from humans to identify differences in cellular responses that might account for pathology.

## 2. Viral Diseases of Bats

Pathogen is a relative term. All zoonotic viruses are, by definition, pathogenic to humans; however, they are usually non- or minimally-pathogenic in the reservoir hosts. Hendra and Nipah viruses do not cause clinical disease in flying foxes [47], and Marburg virus similarly causes only subclinical disease in Egyptian fruit bats [48,49]. Several recent publications have suggested that bats are reservoir hosts of zoonotic viruses because they are somehow special relative to other mammalian species in that they are susceptible to infection but without developing disease [50,51,52]. But there are several infectious diseases of bats, some of which are discussed below, and it is likely that many more as-yet undiscovered infectious diseases affect bats. Moreover, rodent reservoirs of zoonotic viruses remain persistently infected, often for life, without signs of disease, including those that host hantaviruses and arenaviruses [53,54,55,56]. Of those that have been closely examined, a role for regulatory T cells may contribute to persistent infection without disease to these otherwise innocuous viruses [57,58]. Whether similar immune mechanisms occur in reservoir bats that may account for persistence without disease is unknown.

### 2.1. Rabies

It is beyond the scope of this work to adequately discuss rabies virus infections of bats; however, many excellent reviews of rabies virus and other lyssavirus infections of bats have been published [59,60,61]. Few studies have thoroughly examined the host response of bats infected rabies virus. Turmelle *et al.* [62] experimentally infected big brown bats (*Eptesicus fuscus*) with rabies virus and found that most survived for the several months of the study. Moreover, the data suggested that abortive infection did not provide durable immunity in bats and that repeated infections could contribute to resistance to disease. Considering the high densities of wild bat colonies, repeated infections probably occur frequently and may provide a mechanism for resistance to rabies in bats. Persistence of virus in this work may have been linked to low virus replication that failed to stimulate danger signals to the immune response, permitting long-term infection without signs of disease.

Davis *et al.* [63] experimentally infected little brown bats (*Myotis lucifugus*) with rabies viruses and found that intramuscular infection led to rapid clinical progression of rabies, whereas subcutaneous infection led to delayed clinical progression. Notably, bats that developed rabies after subcutaneous infection were more likely to shed virus in salivary glands. Together, the data suggested that route of infection of rabies viruses among bats can influence clinical outcome and potential transmission among bats.

### 2.2. Tacaribe Virus

Tacaribe arenavirus (TCRV) was first identified in two species of artibeus bats in Trinidad and Tobago in the mid-1950s [64]. Wilbur Downs, who was director of the Trinidad Regional Virus Laboratory (TRVL), found a near-dead great fruit-eating bat (*Artibeus literatus*) on the front porch of his home that had been captured by his pet cat [65]. The bat exhibited signs of rabies but subsequent testing excluded rabies because of the lack of Negri bodies. Tissue homogenates of the unidentified agent were inoculated into suckling mice and caused disease. During the next 3 years, 19 isolates of TCRV were made from 6 *A. literatus* and 5 *A. jamaicensis* diseased bats collected on the island of Trinidad; however, only one strain, the original TRVL-11573, remains. Downs proposed that artibeus bats were reservoir hosts of TCRV. However, experimental infection of *A. jamaicensis* with TRVL-11573 resulted in two distinct outcomes; fatal disease with similarities to the South American hemorrhagic fevers within three weeks, or no signs of disease and without substantial viral burden or antibody responses [66]. This work argues against artibeus bats as reservoir hosts of TCRV.

Abraham *et al.* showed that a single amino acid change in human transferrin receptor conferred susceptibility of human cells to Tacaribe virus [67]. Tacaribe virus is closely related to Junín virus and Machupo virus [68], the etiologic agents of Argentine and Bolivian hemorrhagic fevers, respectively. Artibeus bats are found throughout the tropical Americas [69] and it is thought that climate change is expanding their distribution. The bats are currently endemic to the Florida Keys and are expected to expand their range to mainland Florida, where high human population density and agricultural practices (fruit orchards) may provide an opportunity for spillover and emergence into humans. At least one laboratory infection of TCRV has occurred, resulting in flu-like symptoms and fever followed by seroconversion within two weeks [70]. Thus, TCRV has at least some emerging zoonotic disease potential.

### 2.3. Lloviu Virus

In the early 2000s, a large number of Schreiber’s bent-winged bats (*Miniopterus schreibersii*) were found dead in caves in the Iberian Peninsula, the border region of Spain, Portugal and France. Gene sequences of a novel filovirus, Lloviu virus, were detected in all 5 Schreiber’s bats collected (throat and rectal swabs, brain, liver, lung) in a cave near Cantabria, Spain, but in none of 9 dead *Myotis myotis* bats collected at the site [71]. The authors speculated the die-off was a result of Lloviu virus infection; however, without a colony of Schreiber’s bats it has not been possible to fulfill Koch’s postulates.

## 3. Bats as Reservoir Hosts of Important Human Pathogens

The most notable examples of human pathogenic viruses hosted by bat reservoirs are rabies virus and other lyssaviruses, filoviruses, henipaviruses and coronaviruses. Viruses from these groups have had dramatic impacts for the diseases they cause; however, many more viruses also occur in bats and these pose threats as future emerging infectious diseases [22,27,28,72,73]. In each of these bat/virus relationships, the viruses cause little or no harm to the host, or can take months to years to manifest disease [1]. These groups of high-impact agents are RNA viruses and, presumably, the higher error rate of RNA polymerases facilitates virus adaptation to new hosts. For example, rabies virus genotypes are frequently constrained within taxonomic groups of vertebrate hosts (e.g., bat genotypes, fox genotypes), suggesting host biology and/or immunology influences viral evolution [63,74,75].

Hypotheses as to why bats harbor so many infectious agents but without disease have been proposed. One such hypothesis is that elevated metabolic body temperatures from flight, mimics the fever response [51]. Increased body temperature from exertion is a result of consumption of ATP that increases mitochondrial activities that may facilitate host defenses [76] and contribute to reservoir status of bats [52]. However, the fever response that occurs during infection is part of the immunological cascade initiated by infection or inflammation, in which inflammatory cytokines, such as interleukin-1, stimulate the production of prostaglandins that act upon the hypothalamus to increase body temperature [77,78]. By the time fever from an infection occurs, the immune response has already begun in earnest.

## 4. Bat Immune Responses to Viruses

Virtually nothing is known about bat immune responses to viruses. Much of the experimental effort has been conducted with the black flying fox (*Pteropus alecto*), a reservoir of Hendra virus, in the Baker, Wynne and Wang laboratories at CSIRO [2,4,79,80,81,82,83,84,85,86] and recently reviewed in great detail along with other work on bat immunology [87]. As non-model organisms, many obstacles inhibit an understanding of bat immune responses to viruses. Although little experimental work has been performed, substantial genetic and biochemical evidence suggests bats are similar, but not identical, to other mammals in terms of the immunological genes and noncoding RNAs they possess [2,3,4,88], inferring immune responses that are similar to other mammals.

### 4.1. Pattern Recognition Receptors

Nucleated cells have many defensive systems for containment of infectious agents, and many infectious agents encode proteins to circumvent these systems [89,90]. This coevolutionary arms race is largely responsible for shaping host responses and likely has been in effect since the emergence of the first pathogens. Infectious agents often share common biochemical motifs, termed pathogen-associated molecular patterns (PAMP), such as lipopolysaccharides (LPS), lipoteichoic acid, flagellin, or double-stranded RNA that are not present in vertebrates [91]. Consequently, proteins that counteract these PAMPs are highly conserved in vertebrates. The presence of viral PAMPs in cells and tissues is an innate cue that triggers a number of cellular defense mechanisms [92]. All organisms have proteins, termed pattern recognition receptors (PRR), that sense the presence of certain PAMPs and induce amplifying cascades of host cell defense systems, leading to physiologic alteration of the cell and expression of a variety of antiviral genes. Four broad groups of PRRs have been identified in mammals; Toll-like receptors (TLRs), retinoic acid inducible gene-I-like receptors (RLHs), nucleotide-binding oligomerization domain-like receptors (NLRs), and the interferon-inducible absent in melanoma 2 (*Aim2*) [91,93,94].

Many PRRs have been identified, most through scans of genome or transcriptome data that are commonly found in other, well-studied mammals (e.g., humans, laboratory house mice) and suggest bats use these same systems for surveillance of infectious threats. TLR genes have been cloned from two bat species, the black flying fox (*Pteropus alecto*) [95] and Leschenault's rousette bat (*Rousettus leschenaultii*) [96], and are found in genome data from the little brown bat (*Myotis lucifugus*) and transcriptome data from the Jamaican fruit bat (*Artibeus jamaicensis*) [3]. However, there are no reports of activity of these proteins in bats or bat cells infected with viruses. Three RLH genes, *Ddx58* (RIG-I), *Ifih1* (MDA5), *Dhx58* (LGP2), have also been cloned from the black flying fox [80], but no functional studies have been performed with virally-infected bat cells. Three NLR members (*Ciita*, *Nod1*, *Nod2*) are found in some of the bat genetic databases. However, in the two bat species closely examined, *P. alecto* and *Myotis davidii*, the PHYIN locus, which contains the *Aim2* and *Ifit16* genes in other mammals, is absent [4], and orthologous *Aim2* sequences are not found in a transcriptome of *A. jamaicensis* [3].

### 4.2. Interferons

#### 4.2.1. Type I IFN

Multiple type I interferon-α (IFNα) genes exist in mammalian genomes that arose through tandem duplications [97]. *Ifnb* has only a single copy in species that have been examined. These IFN proteins are expressed as downstream events of viral infection and the PRR cascade. Type I interferons have been found in several bat species and infection of bat cells with viruses induces the type I IFN pathway. Virtue *et al.* [82] infected a cell line from *P. alecto* with henipaviruses and found they antagonized the type I IFN response. Biesold *et al.* [98] generated cell lines from the embryonic and adult tissues of several bat species and immortalized them with SV40 large T antigen gene. Cells were infected with Rift Valley fever virus strain 13, which is defective in its type I IFN repression, and found that bat cells expressed *Ifnb* 30-fold higher. Supernatants from these cells exhibited antiviral activities in a VSV reporter system, demonstrating biological activity of bat IFNβ.

*Ifnw* loci are also present in bat genomes and appear to have many tandem copies in species examined, unlike most other mammals [87]. Functional activities of bat IFNω have not been described.

#### 4.2.2. Type II IFN

Interferon-γ (IFNγ) sequences have been identified in two pteropid (*Pteropus alecto*, *P. vampyrus*) and one New World (*Myotis lucifugus*) species [81,87]. *Ifngr1* and *Ifngr2* are found in *A. jamaicensis* suggesting they, too, possess an *Ifng* gene [3]. As with other phylogenetic studies of bat genes, they have some similarity to IFNγ of ungulates. IFNγ from *P. alecto* impairs viral replication in bat cells, reducing Hendra virus replication in *P. alecto* cells and also inhibits Sendai virus replication in a Brazilian free-tail bat (*Tadarida brasiliensis*) lung cell line, indicating broad species cross reactivity [81].

#### 4.2.3. Type III IFN

The type III interferons (IFNλ) are also involved in antiviral responses [99]. Zhou *et al.* [100] produced recombinant *P. alecto* IFNλ and examined its ability to induce *Ifit1* (Isg56) and *Ddx58* (RIG-I), and to interfere with Tioman virus-mediated cell lysis. Ifit1 recognizes 5’ triphosphate-RNA produced by single-stranded RNA viruses. They found elevated IFNλ expression in *P. alecto* splenocytes infected with Tioman virus. IFNλ also inhibited Pulau virus replication and dramatically increased expression of *Ifit1* and, to a lesser extent, *Ddx58* in a *P. alecto* cell line. IFNβ and IFNλ induced expression of P. alecto Mx1, a GTPase that may target viral nucleoproteins, and Oas1, which activates RNaseL and degradation of viral RNA, but not *Pkr* [86], and that *P. alecto* splenocytes are highly sensitive to IFNλ stimulation [100]. It is likely that bat type I and type III IFNs induce expression of many other antiviral genes and, considering their properties, that viruses naturally hosted by bats have countermeasures that target proteins of the IFN pathways.

### 4.3. Responses of Innate Immune Cells and Lymphocytes

Specialized immune cells play essential roles in the host response to infection. Several dendritic cell subsets, macrophages, neutrophils and NK cells are responsible for early containment of infectious threats. While morphological descriptions of some of these cells and genomic/transcriptomic evidence of their existence in bats have been made, no work has been performed to examine responses of these bat cells to viruses.

Bats also have T and B cells as evidenced by cellular purification, the production of high titered IgG [101,102,103,104] (which requires T cell help) and sequences in the various bat genome and transcriptome datasets. However, only antibody responses have been examined by serological assays. No *in vitro* or *ex vivo* work on antigen-specific T cells or B cells have been reported. A significant limitation of conducting research into bat lymphocyte responses to viruses is the lack of closed colonies for experimental infection work, suitable growth factors for expansion of antigen-specific lymphocytes (e.g., IL-2, IL-4, GM-CSF, Flt3L, *etc.*), and the methodologies for bat lymphocyte culture.

IgG isotypes and copy number vary between bat species but exhibit evidence of substantial diversity [79,105]. Only a single IgG isotype has been detected in *Carollia perspicillata*, whereas *Eptesicus fuscus* appears to have two IgG isotypes, and *Myotis lucifugus* has five IgG isotypes [106]. Another unusual feature of *M. lucifugus* is that it has a very high diversity of VDJH locus but little evidence of somatic hypermutation. This may represent an evolutionary alternative for generation of antibody diversity. It is unknown if these differences have immunological significance.

## 5. Approaches for Studying Bat Immune Responses

Many significant challenges for understanding immune responses of bats exist. A lack of closed colonies, the genetic diversity of bats (between and within species), few immunological, molecular or biochemical reagents and cell lines, the large number of bat species and their viruses, and the difficulty of working with bats all contribute to these challenges. However, novel technologies and instrumentation that are substantially species-independent are now available, particularly RNA-Seq and metabolomics, that can facilitate the rapid discovery of bat-virus interactions. Proteomics tools are substantially limited because many of these require ligand-specific antibodies to target proteins that are largely unavailable for bats; however, combined use of transcritome and liquid chromatography/mass spectrometry analysis may help circumvent this limitation [84,107]. Some antibodies are cross reactive with bat proteins, but most appear to be towards highly conserved intracellular proteins such as those involved in the antiviral response. Fujii *et al.* showed that antibodies to STAT1 and pSTAT1^Y701^ are cross reactive with *Rousettus aegyptiacus* STAT1 [108], and these antibodies may be useful for examining how Marburg virus VP40 interferes with Egyptian fruit bat STAT1 activities [109]. Considering STAT1’s conservation, it is likely these antibodies are also cross-reactive with STAT1 from other bat species.

### 5.1. Husbandry

**Figure 1:**
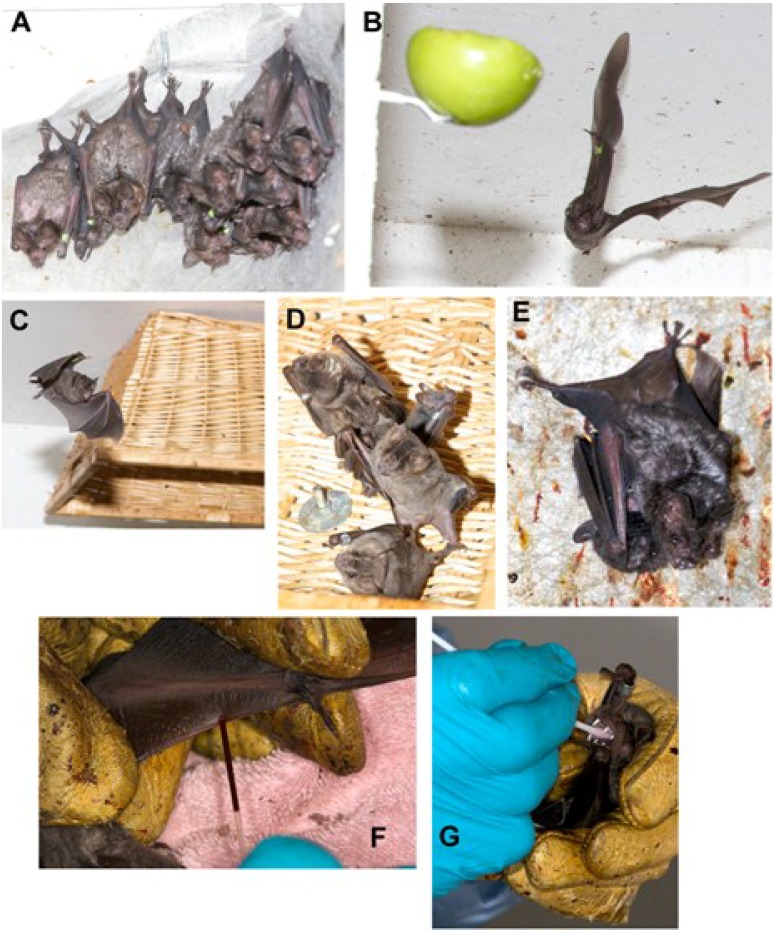
Bat husbandry and manipulations. Establishment of bat colonies for experimental research requires enrichment for successful maintenance and reproduction. (**A**) Landscape fabric attached to walls provides roosting substrate; (**B**) Hanging of fruit on skewers can provide enrichment for frugivorous bats by stimulating foraging behavior. A wing band (green) is present with a unique identification number; (**C**, **D**) Hanging of inverted wicker or metal baskets provide additional roosting substrates; (**E**) A female Seba’s fruit-eating bat (*Carollia perspicillata*) with offspring; (**F**) Blood collection from a bat wing; (**G**) Oral swab for virus collection. Photos are by the author and are of the colony at the University of Northern Colorado [3,66,106].

Few breeding colonies are available for use in bat research, principally because of a lack of sources for obtaining bats, expertise in veterinary care, adequate facilities and the cost of maintaining colonies. Most zoos and zoological organizations are unwilling to provide bats for biomedical research purposes, and this policy has significantly stymied research into bat immunology and impairs understanding of infectious diseases that kill bats, including white nose syndrome. The establishment of closed breeding colonies for research purposes is an essential component for developing controlled, hypothesis-driven research (Figure 1).

Free-flight rooms are highly desirable for welfare and, perhaps required, for successful breeding colonies of most bat species. The ability to fly in an enclosure is probably necessary for the social health of the bats and will likely improve reproductive success. The decision of which bat species to use is driven by the virus-of-interest, and husbandry requirements are frequently specialized. In general, fruit bats are easier to colonize than are insectivorous bats, which must learn to eat non-flying insects. Enrichment is also necessary for the welfare of colonized bats, including artificial roosts, hanging of fruit from walls or ceilings to stimulate foraging behavior, and places to hide, such as landscape fabric hanging from a wall.

For experimental infections, bats may require housing in cages that may prevent flight. Many of the viruses of interest hosted by bats require BSL-3 or BSL-4 containment, which limits the number of institutions where such work can be performed. Despite these difficulties, managing colonies of certain species of bats for research purposes is readily attainable.

### 5.2. RNA-Seq

Deep sequencing approaches [110] for examination of transcriptional profiles of bats and bat cells infected with viruses can shed light on biochemical pathways that are activated or repressed, thus inform about the host response and potential cellular targets of viral evasion. The costs of RNA sequencing (RNA-Seq) have become considerably less in the last several years. Improvements in instrumentation have increased depth of coverage, making the price per gigabase even less expensive. The cost of cDNA library construction has not substantially improved, but can be done for about US$100 per sample. Moreover, preparation of RNA and libraries for nuclear gene transcripts, noncoding RNAs (e.g., miRNA) and viruses required different methodologies. Transcripts of some genes that encode potent proteins, such as cytokines, are often expressed at very low levels and have short half-lives [111] that can make their detection difficult or impossible, even with deeper coverage that is more expensive. For these genes, real-time PCR is necessary to identify differences in gene expression. Nonetheless, RNA-Seq offers investigators a powerful tool to generate large amounts of data, regardless of the bat species of interest.

A significant complicating factor of RNA-Seq is the requirement for substantial computational resources and expertise. Assembly of reads from vertebrate RNA-Seq data requires multiprocessor/ multicore computers, hundreds of gigabytes of RAM, terabytes of hard drive capacity and familiarity with Linux operating systems. While many institutions have such computational resources, others do not. Fortunately, two such resources that use Trinity (described below) are publicly available; the Pittsburg Supercomputer Center (https://www.psc.edu) and the Data Intensive Academic Grid (http://www.diagcomputing.org/). Thus, the lack of computational resources by research groups is not a significant obstacle so long as internet access is available for data transfer and a willingness to wait for sample processing because queue times can be long.

In general, when working with a species that does not have an annotated genome available it is necessary to generate *de novo* assemblies of all the RNA-Seq sample data from an experiment. By doing so, one ensures that every transcript and isoform is represented in the assembled dataset. Several RNA-Seq assembly programs are available in the public domain, including Trinity [112] and Oases [113]. These programs are designed for RNA assembly; other applications are available for DNA assembly. Each program has advantages and disadvantages, so it is essential to carefully examine packages to determine which is most suited for a given dataset. Once the *de novo* assembly is completed, other software tools are employed to count reads from each sample for use in statistical analysis of transcript and isoform abundances. Several abundance estimation packages are freely available, including the Bioconductor package (composed of edgeR and DESeq) [114,115], and RSEM [116] and eXpress annotation [117] that provide quantitative and qualitative statistical analysis of differentially-expressed genes.

**Figure 2:**
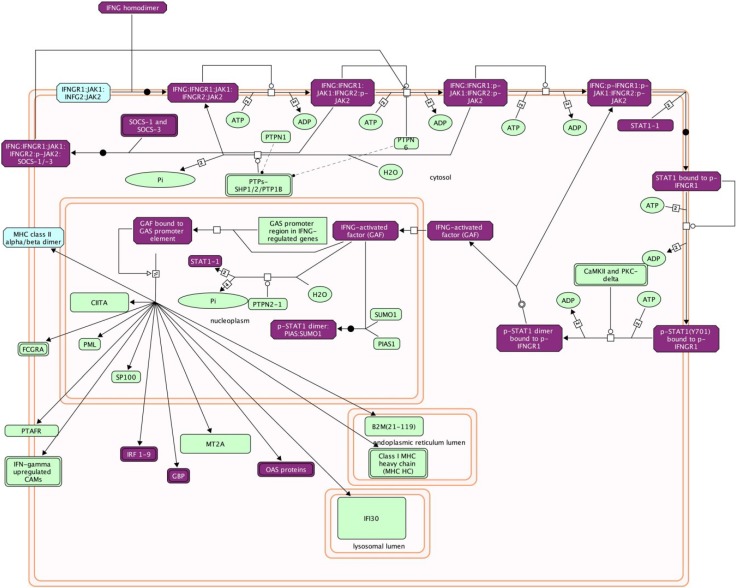
Reactome mapping of differentially-expressed genes. Abundance of differentially-expressed IFNγ pathway transcripts in Pirital arenavirus-infected hamster livers relative to uninfected group were estimated by RSEM and statistically analyzed with DESeq. The symbols for the elevated genes were imported into the Reactome plugin of Cytoscape for pathway analysis. Genes in purple boxes were elevated in infected animals (author’s unpublished data) and suggests this pathway is important in the host response to the virus.

It is not uncommon for more than a thousand genes to be differentially expressed in an experiment and identification of those transcripts is required for subsequent analysis. Batch BLAST is used to identify transcripts using the RefSeq_RNA, mir-Base and IgProt databases, and many BLAST tools are available, including web-based, command line and GUI programs that are free or commercial. Most transcripts will be identified by BLAST, including some noncoding RNAs; however, many others will have no hits in the databases and these transcripts can be difficult to interpret. Determining the roles of differentially-expressed genes can be readily identified using DAVID [118] and Reactome [119] (remotely or from within Cytoscape software [120]). These applications provide pathway information about proteins, including other proteins in those pathways (Figure 2). These packages are essential for rapidly identifying potential viral targets during infection and can suggest which pathways should be further scrutinized.

### 5.3. Real-Time PCR

Although RNA-Seq can provide broad information about expression of genes and pathways that may be important in host responses to viruses, it is limited in its ability to identify genes expressed in very low abundances. Real-time PCR is a mature technology that can be more sensitive than RNA-Seq and is rapidly deployable for multiplex examination of gene expression [46,121]. However, because few bat genome and transcriptome datasets are available, obtaining sequences of genes of interest can be challenging. Some immune genes are highly conserved (e.g., *Tnf*) whereas others are less so (e.g., *Ifng*). Because RNA-Seq often misses these transcripts (because their abundances are too low or were not expressed in the tissue from which the RNA was extracted) it may not be suitable for obtaining sequence data for developing primer sets for genes of interest. There are other approaches for obtaining sequences of unknown genes, principally degenerate PCR using primers designed from orthologous sequences; however, this approach often fails to amplify specific products. Sequencing of genomes is one approach to mitigating this problem and its cost has also decreased substantially. The principal advantage of genome sequencing is that all genetic information is essentially present in datasets, unlike RNA-Seq where many genes are not detected. New tools are now available that can rapidly mine unannotated genome (and transcriptome) data that performs *in silico* assembly of cDNA sequences for automated primer design [121]. With sufficient genome sequencing coverage, this approach identifies nearly all genes, many of which may be absent from RNA-Seq data.

### 5.4. Metabolomics

Metabolomics is a recently-emerging field that can provide insight into biochemical activities during infections and immune responses, and is based upon the detection of specific metabolites [122,123,124]. For bat research, this can provide valuable information because many metabolites are identical or similar, regardless of species. Because the biochemical pathways for producing these metabolites are often conserved among species, profiling provides information on host metabolic responses to viral infections. Some viruses remodel the metabolism of infected cells for the production of progeny that may contribute to pathogenesis. For example, hepatitis C virus alters phospholipid and sphingomyelin abundance and leads to elevated ceramides, which are toxic to hepatocytes [125]. These lipids are readily detected by metabolic profiling and such approaches may be applied to studies of bats and their viruses.

### 5.5. Immune Cell Culture

**Figure 3:**
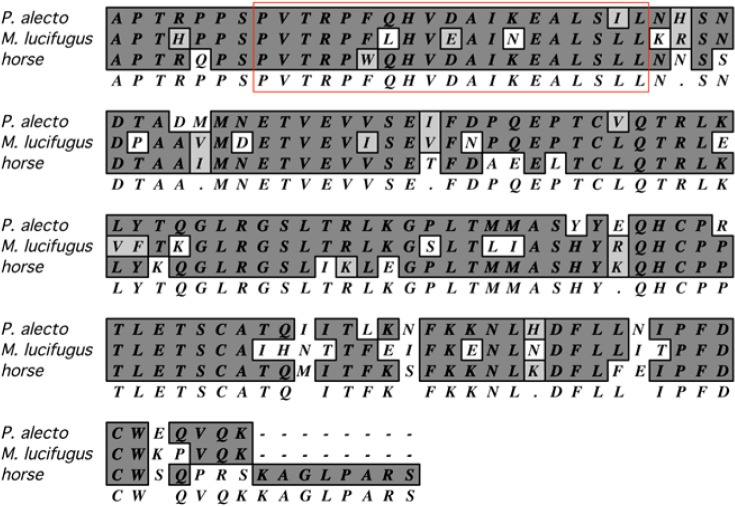
Amino acid alignment of mature GM-CSF from horse, *P. alecto* and *M. lucifugus*. Identical amino acids in dark gray, similar amino acids in light gray, and dissimilar amino acids in white, consensus below. The red box is the putative helix A involved in binding to the GM-CSF receptor. Alignment performed with MacVector using the default CLUSTAW settings [128].

Substantial information about immune responses can be garnered from studies of antigen-specific T and B cells. However, several obstacles impede such research, including the lack of recombinant bat cytokines for the growth and propagation of immune cells and the lack of MHC-matched, syngeneic animals and cell lines for T cell work. It may be that commercially-available reagents for species that are phylogenetically closer to bats, such as those for horse and cattle, may be used for establishing long-term antigen-specific cultures of bat T and B cells. In particular, helper T cell lines require periodic restimulation with antigens presented on syngeneic MHC class II proteins found on antigen presenting cells (APC), and a requirement of IL-2. Without inbred bats, it is difficult to conduct such work. One solution to this problem may be the use of autologous APC, and bone marrow is a rich source of progenitor cells for their generation. Granulocyte-macrophage colony stimulating factor (GM-CSF) is routinely used for generating common dendritic cells (cDC) that are efficient APC for helper T cells [126,127]. Horse GM-CSF is commercially available and shares substantial similarity to orthologous GM-CSF in several bat species, including the receptor-binding domain (Figure 3), and may be useful for propagation of cDC from bat bone marrow.

The humerus and radius bones of bats are rich sources of bone marrow and are easily prepared for bone marrow retrieval (author’s unpublished observation). The small sizes of many bat species presents a challenge for obtaining large amounts of bone marrow; however, many bat species of interest for zoonotic diseases (e.g., pteropid, rousette) weigh more than a hundred grams and it should be possible to obtain large numbers of bone marrow cells that can be aliquoted and stored frozen for long-term use for T cell antigen recall experiments.

## 6. Conclusions

Although many difficulties and obstacles exist for conducting hypothesis-driven, experimental infection research of bats and their viruses, new technological advancements can permit rapid discovery of their relationships. However, before substantial progress can be made, closed colonies of specific pathogen-free bats need to be established. Field studies are of limited value for examination of the fine granularity of reservoir host responses to viruses, and the capture of wild bats for experimental infection experiments has a risk of infectious and environmental factors that can complicate interpretation of data. Susceptible bat cell lines and antibodies for use with bat proteins must be identified or generated for closer scrutiny of how these viruses interact with their host cell machinery, and how they might manipulate the host response in a manner that is favorable for both the virus and the host. Until then, it will be nearly impossible to answer the question: Are bats special?
